# Supplementary figures and images for: Expanding radiogenic strontium isotope baseline data for central Mexican paleomobility studies
Source: PLoS One. 2020 Feb 24;15(2):e0229687. doi: 10.1371/journal.pone.0229687 (PMC7039465; doi:10.1371/journal.pone.0229687)

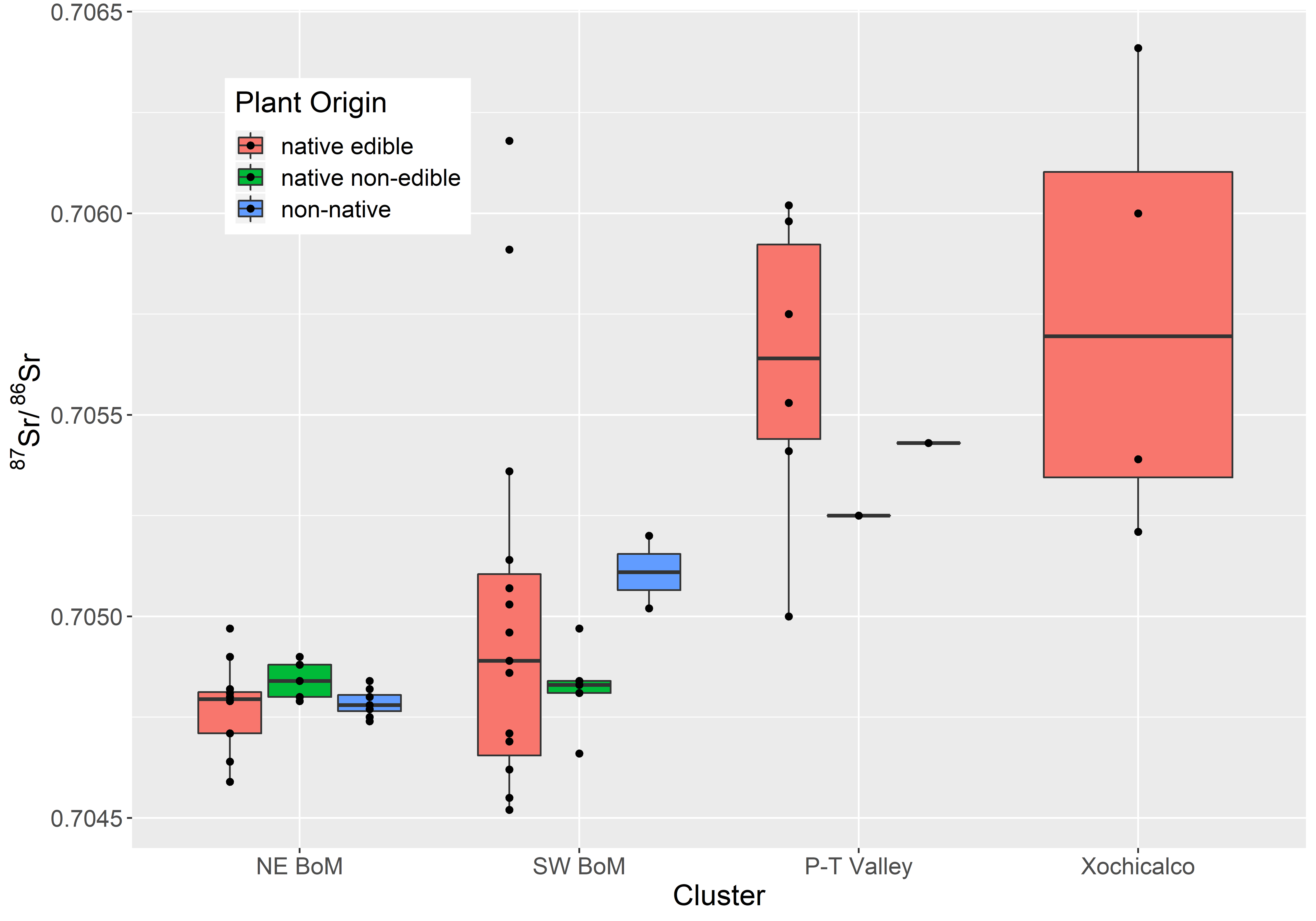

Supplement: S1 Fig — There were no significant differences between edible native plants and non-edible native plants or non-native plants. While non-edible native and non-native plants would not have contributed to past human and animal bioavailable 87Sr/86Sr values, they are included in this study to further characterize bioavailable strontium values in local ecosystems. (TIF) [file pone.0229687.s003.tif]
