# Supplementary material for: Expanding radiogenic strontium isotope baseline data for central Mexican paleomobility studies
Source: PLoS One. 2020 Feb 24;15(2):e0229687. doi: 10.1371/journal.pone.0229687 (PMC7039465; doi:10.1371/journal.pone.0229687)
Supplement: S1 Table — (DOCX) [file pone.0229687.s006.docx]

**S1 Table. Central Mexican site-level ^87^Sr/^86^Sr medians and interquartile ranges.**

| Cluster | Site | Median  ^87^Sr/^86^Sr | Interquartile ^87^Sr/^86^Sr Range | | *n* |
| --- | --- | --- | --- | --- | --- |
| 1 | TQX | 0.70466 | 0.70461 - | 0.70471 | 4 |
| 1 | TUL^a^ | 0.70500 | 0.70485 - | 0.70501 | 3 |
| 2 | TEO^a,b^ | 0.70459 | 0.70441 - | 0.70465 | 13 |
| 2 | TZG | 0.70464 | 0.70462 - | 0.70468 | 3 |
| 2 | XAL | 0.70480 | 0.70478 - | 0.70484 | 22 |
| 3 | ATO | 0.70481 | 0.70467 - | 0.70484 | 3 |
| 3 | CMZ | 0.70471 | 0.70463 - | 0.70480 | 3 |
| 3 | CUI | 0.70522 | 0.70506- | 0.70550 | 4 |
| 3 | NAU | 0.70497 | 0.70483 - | 0.70500 | 3 |
| 3 | TEZ^c^ | 0.70569 | 0.70520 - | 0.70618 | 2 |
| 3 | TLC | 0.70490 | 0.70484 - | 0.70501 | 4 |
| 3 | TYH | 0.70462 | 0.70459 - | 0.70464 | 3 |
| 4 | CHL | 0.70587 | 0.70567 - | 0.70599 | 4 |
| 4 | CXT | 0.70533 | 0.70519 - | 0.70544 | 4 |
| 5 | XCL | 0.70570 | 0.70535 - | 0.70610 | 4 |

^a^From data on bulk soil samples published in Schaaf et al. [15]

^b^From data on faunal remains published in Price et al. [12]

^c^Only two samples were analyzed from Tezozomoc, so a simple range is presented rather than an interquartile range.
