# Supplementary material for: Expanding radiogenic strontium isotope baseline data for central Mexican paleomobility studies
Source: PLoS One. 2020 Feb 24;15(2):e0229687. doi: 10.1371/journal.pone.0229687 (PMC7039465; doi:10.1371/journal.pone.0229687)
Supplement: S1 Translation — (DOCX) [file pone.0229687.s007.docx]

Ampliando valores de referencia de isótopos radiogénicos de estroncio para estudios de la paleomovilidad en el centro de México

Sofía I. Pacheco-Forés^1*^, Gwyneth W. Gordon^2^, y Kelly J. Knudson^1^

^1^Center for Bioarchaelogical Research, School of Human Evolution and Social Change, Arizona State University, Tempe, AZ, United States of America

^2^School of Earth and Space Exploration, Arizona State University, Tempe, AZ, United States of America

* Autora correspondiente

Email: [sipachec@asu.edu](mailto:sipachec@asu.edu) (SIPF)

# Resumen

Los isótopos de estroncio radiogénico (^87^Sr/^86^Sr) se han utilizado por mucho tiempo en análisis de paleomovilidad en Mesoamérica. Aunque se ha dedicado un esfuerzo considerable al desarrollo de valores de referencia ^87^Sr/^86^Sr en toda la región maya, el trabajo en el centro de México se enfoca principalmente en el centro urbano del período Clásico de Teotihuacán. Este estudio se suma a este importante conjunto de datos al presentar valores de ^87^Sr/^86^Sr biodisponibles en todo el centro de México, centrándose en la Cuenca de México. Por lo tanto, este estudio sirve para ampliar la utilidad de los isótopos de estroncio en una región geográfica más amplia. Se recolectaron 63 muestras de plantas y agua de 13 sitios del centro de México y se analizaron para ^87^Sr/^86^Sr en un espectrómetro de masas de plasma acoplado inductivamente de múltiples colectores Thermo-Finnigan Neptune (MC-ICP-MS). Estos datos se analizaron junto con 16 valores ^87^Sr/^86^Sr publicados de dos sitios adicionales dentro de la región de interés. Luego se generó un modelo de k-means de cinco grupos para determinar cuáles regiones en la Cuenca de México y del centro de México se pueden distinguir isotópicamente utilizando los valores ^87^Sr/^86^Sr. Aunque los dos grupos que caen dentro de la Cuenca de México se superponen en sus rangos locales de ^87^Sr/^86^Sr, muchos sitios dentro de la Cuenca se distinguen utilizando los valores ^87^Sr/^86^Sr a nivel de sitio. Este estudio contribuye a los estudios de paleomovilidad en el centro de México al expandir el conocimiento de la variabilidad de los isótopos de estroncio dentro de la región, lo que finalmente permite a los investigadores detectar la movilidad residencial intrarregional y obtener una mayor comprensión de las interacciones sociopolíticas entre la Cuenca de México y regiones periféricas del centro de México.

Palabras claves: isótopos de estroncio radiogénico, Cuenca de México, historia residencial, biogeoquímica

# Introducción

Los investigadores han debatido durante mucho tiempo la importancia de la migración en el desarrollo cultural del centro de México. Varios análisis arqueológicos [1-4], morfológicos [5-9] y genéticos [10,11] indican que la Cuenca de México atrajo múltiples oleadas de migrantes de toda la Gran Mesoamérica durante la época prehispánica. Los estudios biogeoquímicos de isótopos de estroncio radiogénico (^87^Sr/^86^Sr) han demostrado ser efectivos para comprobar directamente la presencia de migrantes dentro de la Cuenca, particularmente en la ciudad del período Clásico de Teotihuacán [12-17]. Aunque es esencial determinar rangos de variación “locales” en valores de ^87^Sr/^86^Sr para la aplicación adicional de este método, los datos de estroncio radiogénico del centro de México fuera de Teotihuacán son escasos. Price y colegas [18] establecieron un rango regional de ^87^Sr/^86^Sr para la Cuenca de México, pero pocos estudios examinan la variabilidad dentro de la Cuenca o el centro de México.

Este estudio investiga la variabilidad del estroncio radiogénico dentro de la Cuenca de México y el centro de México, facilitando aún más los estudios de paleomovilidad dentro de la región. Primero discutimos el uso de isótopos de estroncio en la paleomovilidad dentro de Mesoamérica y más allá y luego consideramos las expectativas geológicas para los valores de ^87^Sr/^86^Sr en el centro de México y la Cuenca de México. Finalmente, presentamos datos biogeoquímicos en muestras modernas de plantas y agua (*n*=63), analizándolos junto con datos publicados (*n*=16) [12,15] para caracterizar zonas biogeoquímicamente distinguibles dentro de la Cuenca de México y el centro de México.

# Isótopos de estroncio en estudios de paleomovilidad

Los isótopos de estroncio radiogénico son uno de varios sistemas isotópicos que se han utilizado para caracterizar la paleomovilidad [19-23]. Los valores de ^87^Sr/^86^Sr reflejan la variabilidad geológica regional [24]. El estroncio biológicamente disponible presente en el suelo y las aguas subterráneas se incorpora a las plantas locales y posteriormente a la hidroxiapatita, los tejidos duros que incluyen los huesos y el esmalte de los animales que ingieren esa vegetación [25-28]. Al comparar los valores isotópicos de estroncio en los tejidos duros humanos y animales que se mineralizan en diferentes momentos durante el curso de la vida, los bioarqueólogos pueden reconstruir patrones prehistóricos de movilidad entre distintas zonas geológicas a lo largo del curso de la vida [12,20,29-31].

## Sistemática de isótopos de estroncio

El estroncio es un metal alcalinotérreo que se encuentra típicamente en roca, agua, suelo, plantas y animales en el nivel de partes por millón (ppm) [24,32]. De los cuatro isótopos de estroncio naturales, el ^87^Sr es radiogénico y se produce por la lenta descomposición radiactiva del rubidio (^87^Rb). Por lo tanto, la abundancia de ^87^Sr en una región varía según la edad y la composición de los minerales del lecho rocoso local [24,32]. Las formaciones ígneas y graníticas geológicamente más antiguas ricas en ^87^Rb padre se enriquecen en ^87^Sr (^87^Sr/^86^Sr> 0.750) en comparación con los basaltos, riolitas o andesitas volcánicas geológicamente más jóvenes (^87^Sr/^86^Sr≈0.702-0.704), mientras que los carbonatos marinos y las formaciones metamórficas a menudo tienen valores intermedios [24,33,34]. Existe un amplio rango de variación en comparación con el error instrumental de las mediciones del espectrómetro de masas, que puede generar mediciones precisas hasta el cuarto decimal o mejor (± 0.00001) [31,34]. Como tal, se pueden usar mapas geológicos de tipos y edades de roca de fondo para predecir la variación esperada de ^87^Sr/^86^Sr.

Sin embargo, las predicciones basadas exclusivamente en mapas geológicos de tipos de roca firme no siempre son precisas. Se pueden mezclar una serie de factores, incluyendo a la modificación de la roca fuente por la erosión y la meteorización preferencial del mineral con valores más radiogénicas, la adición de material derivado del viento y la pulverización marina. Estos factores pueden producir diferentes proporciones de estroncio biodisponibles que finalmente terminan incorporado en hidroxiapatita [34-36]. Por lo tanto, los investigadores han realizado estudios de isótopos de estroncio de fuentes de agua locales, suelos, plantas y huesos de animales para caracterizar con mayor precisión la variabilidad del estroncio biodisponible en un entorno determinado [28,37-43].

## Isótopos de estroncio y paleomovilidad en Mesoamérica

Los estudios que utilizan los isótopos ^87^Sr/^86^Sr para reconstruir la paleomovilidad en toda Mesoamérica han aumentado dramáticamente en los últimos años a medida que los arqueólogos buscan comprobar hipótesis de migración, diáspora y movilidad antiguas dentro de la región directamente [44]. Investigadores han utilizado isótopos de estroncio radiogénicos para reconstruir patrones de migración antiguos [12,17,41,45-50], los orígenes geográficos de las víctimas de sacrificio [13,51] y las redes de gestión y comercio de animales en el pasado [52,53], así como redes de comercio de cultura material de larga distancia [54] y diásporas históricas [55,56].

Otros estudios se han centrado en caracterizar la variabilidad ^87^Sr/^86^Sr en Mesoamérica. Hodell y colegas [40] llevaron a cabo un extenso estudio sobre la variabilidad del estroncio radiogénico en la región maya del sur de México, Belice y Guatemala para identificar subregiones isotópicamente distintas. De manera similar, Price y colegas [18] analizaron los rangos ^87^Sr/^86^Sr de manera más amplia en Mesoamérica. Aunque informan un rango local de ^87^Sr/^86^Sr= 0.7046-0.7051 para la Cuenca de México, existen pocos datos publicados que examinen la variabilidad dentro del centro de México y la Cuenca misma.

# Geografía, geología y geoquímica del centro de México

El conocimiento de la geología regional es esencial para el estudio de la variabilidad en los valores de isótopos de estroncio radiogénicos en el centro de México, que se define aquí como la inclusión de los estados mexicanos modernos del Estado de México, Hidalgo, Puebla, Tlaxcala y Morelos, así como la Ciudad de México. Los geólogos han dividido a México en varias provincias morfotectónicas que son geológicamente y fisiográficamente distintas (Fig 1). Sin embargo, solo tres provincias morfotectónicas, la Sierra Madre Oriental, el Arco Volcánico Transamericano y la Sierra Madre Sur, conforman el centro de México.

**Fig. 1. Las provincias morfotectónicas de México.** El centro de México está delineado en rojo y está formado por partes de las provincias morfotectónicas de la Sierra Madre Oriental (5), el Arco Volcánico Transamericano (8) y de la Sierra Madre del Sur (9). Otras provincias morfotectónicas incluyen la península de Baja California (1), las llanuras y sierras del noroeste (2), la Sierra Madre Occidental (3), las mesetas y cordilleras Chihuahua-Coahuila (4), la llanura de la costa del Golfo (6), la meseta central (7), la Sierra Madre de Chiapas (10) y la Plataforma de Yucatán (11). Los sitios incluidos en el estudio se indican con puntos negros. Mapa creado por SIPF con datos de mapas vectoriales y rasterizados de Natural Earth [57]. Datos morfotectónicos adaptados del Servicio Geológico Mexicano [58].

La geología del centro de México es una mezcla compleja de tierras altas volcánicas recientes y depósitos sedimentarios marinos más antiguos, junto con una variedad de rocas metamórficas [59-61]. La zona norteña del centro de México se compone de la cordillera de la Sierra Madre Oriental que se compone principalmente de carbonatos, areniscas y lutitas sedimentarias mesozoicas, jurásicas y cretáceas orogénicas de origen marino con algunos gneis metamórficos precámbricos y paleozoicos y afloramientos de esquisto [61,62]. Inmediatamente al sur y formando el corazón del centro de México se encuentra el Arco Volcánico Transamericano, que se extiende desde las costas del Pacífico hasta el Golfo. El Arco Volcánico Transamericano es una meseta volcánica cenozoica con andesitas basálticas centrales que se formaron durante el Mioceno tardío y el Plioceno temprano y las andesitas, dacitas y riolitas del sur más jóvenes que se formaron más recientemente durante el Cuaternario [59,61,63-65].

Finalmente, el extremo sur del centro de México está definido por la cordillera de la Sierra Madre del Sur. La Sierra Madre del Sur es la provincia morfotectónica más geológicamente compleja de México. Se compone por un segmento norteño de sedimentos mesozoicos jurásicos y cretáceos y afloramientos de roca volcánica parcialmente cubiertos por rocas volcánicas y sedimentarias cenozoicas. En la zona sureña de la provincia se encuentran afloramientos de roca metamórfica paleozoica y mesozoica e intrusivos batolitos mesozoicos y cenozoicos. La zona costanera del Pacífico se compone de rocas volcánicas sedimentarias jurásicas y cretáceas mesozoicas andesíticas [60,61].

## La Cuenca de México en contexto geológico

La Cuenca de México, la región de principal interés en este estudio se localiza en la parte centro-oriental del Arco Volcánico Transamericano. Es una cuenca graben terciaria y cuaternaria tardía caracterizada por un volcanismo basáltico y andesítico con conos de riolita individuales, presentando algunas de las geologías volcánicas más complejas de México [61,63,66,67]. La Cuenca está rodeada por varias cordilleras, incluidas la Sierra de Tepotzotlán y la Sierra de Pachuca al norte, la Sierra de Río Frío y la Sierra Nevada al este, la Sierra de Chichinautzin al sur, y la Sierra del Ajusco y Sierra de las Cruces al oeste.

Aunque la geología subyacente de la roca firme es probablemente la contribución dominante a la composición de isótopos de estroncio radiogénico del piedemonte y las montañas de la Cuenca de México, la llanura aluvial representa una gran área de captación de minerales erosionados depositados por ríos y arroyos que fluyen hacia los lagos de la Cuenca. En elevaciones altas, que tienden a tener altas velocidades de meteorización, los valores de ^87^Sr/^86^Sr en la roca firme y los valores de ^87^Sr/^86^Sr biodisponibles se correlacionan más a menudo [68,69]. Sin embargo, en elevaciones más bajas, las correlaciones entre la roca firme subyacente y el contenido del río son menos claras, ya que los ríos transportan cargas suspendidas de rocas y sólidos río arriba, así como la precipitación, todo lo cual podría contribuir valores de estroncio que son geológicamente distintos a los depósitos aluviales [37,40,70]. Esto sugiere que los suelos en la llanura aluvial de la Cuenca de México pueden variar considerablemente en valores de isótopos de estroncio y probablemente promediarán los materiales de origen. Por lo tanto, aunque la geología de la Cuenca de México proporciona expectativas iniciales para los rangos de variabilidad del estroncio radiogénico, es necesario generar rangos "locales" esperados de valores de estroncio biodisponibles dentro de la región para obtener una comprensión más integral de la variabilidad dentro y más allá de la Cuenca de México.

# Materiales y métodos

## Recopilación de muestras

Las muestras modernas de plantas y agua proporcionan un medio excelente para caracterizar el estroncio biodisponible dentro de los ecosistemas. Aunque que los valores ^87^Sr/^86^Sr de muestras de suelo en una zona geológica pueden variar mucho debido a las distintas concentraciones de estroncio y a los perfiles de meteorización de los minerales en el lecho de roca subyacente [34,71], solo una proporción del estroncio del suelo está disponible para las plantas. Como tal, los valores ^87^Sr/^86^Sr de las plantas proporcionan un promedio constante de estroncio biodisponible local dentro de un ecosistema [72]. Del mismo modo, la mayoría del estroncio en las fuentes de agua se transporta como sedimento disuelto o suspendido y representa principalmente el estroncio biodisponible de las rocas que sufren erosión dentro de un ecosistema [34,37,69,70,73,74].

Se recolectaron muestras de plantas y agua entre diciembre de 2015 y junio de 2017 de un total de 13 sitios arqueológicos y agrícolas de distintas zonas ecológicas en toda la Cuenca de México y el centro de México (*n*=63). Los datos de elevación y coordenadas de Universal Transverse Mercator (UTM) para cada muestra se recolectaron utilizando una unidad de GPS portátil (S1 File). Las muestras de plantas solo se recolectaron si estaba claro que no habían sido tratadas con fertilizantes o agua de riego, ya que esto podría sesgar los valores de estroncio biodisponible local con fuentes no locales de estroncio. Además, se tomaron muestras de plantas de profundidades de enraizamiento variadas de manera oportunista. Las plantas con raíces poco profundas en la capa superficial del suelo (<1 m de profundidad), como los pastos y muchas plantas herbáceas, tienden a exhibir valores de ^87^Sr/^86^Sr más cerca del polvo atmosférico. Por el contrario, las plantas con profundidades de enraizamiento más profundas, incluidas muchas especies de árboles, exhiben valores de ^87^Sr/^86^Sr derivados de la roca madre local además de las fuentes atmosféricas [36]. La inclusión de ambas fuentes permite una caracterización más precisa del estroncio biodisponible en los ecosistemas locales [75]. Del mismo modo, las muestras de agua solo se recolectaron de manantiales no contaminados que probablemente habrían sido utilizados por antiguos habitantes de la región [76,77]. El Instituto Nacional de Antropología e Historia (INAH) no requiere permisos específicos para recolectar agua o muestras de plantas modernas de los sitios en el estudio. Además, no se incluyeron especies de plantas en peligro de extinción o protegidas en el estudio. Las muestras se importaron al Archaeological Chemistry Laboratory de Arizona State University bajo permisos otorgados a Pacheco-Forés del United States Department of Agriculture Animal and Plant Health Inspection Service (PCIP-17-00469).

Además, los valores publicados de ^87^Sr/^86^Sr en México central generados por Price y colegas [12] y Schaaf y colegas [15] se incluyeron en el conjunto de datos del estudio (*n*=16). Se incorporaron muestras de referencia no humanas como suelos, plantas o materiales de fauna [37]. No se incluyeron datos de muestras de rocas enteras publicadas, ya que estos valores ^87^Sr/^86^Sr probablemente no estaban biodisponibles dentro del ecosistema. Finalmente, los datos publicados se incluyeron solo si su procedencia podía confirmarse mediante GPS para proporcionar datos de elevación y coordenadas UTM razonablemente precisos.

## Métodos biogeoquímicos

Todas las muestras fueron preparadas en el Archaeological Chemistry Laboratory de Arizona State University. Las muestras de agua se filtraron (2,5 m de diámetro) y se acidificaron hasta 5% HCl para evitar la formación de precipitados, la adsorción a las paredes de la botella y desalentar el crecimiento de bacterias y algas. Cuando posible, los componentes de las dietas prehispánicas se simularon mediante el aislamiento manual y el análisis de componentes comestibles (e.g., semillas, bayas, hojas) de plantas secas [78]. Las muestras de plantas se enjuagaron con agua Millipore 18,2 MΩ para eliminar la suciedad adherida y se incineraron en un horno durante aproximadamente 10 horas a 800ºC. Se digirieron aproximadamente 25,0 mg de muestra de ceniza en 2 ml de ácido nítrico y clorhídrico concentrado (HNO_3_ + 3HCl) a aproximadamente 50°C durante 24 horas. Esta lixiviación agresiva no descompone la estructura de tetraedros de sílice de la mayoría de los minerales de silicato, dejando gran parte del suelo en forma sólida y priorizando la liberación de estroncio biodisponible dentro de las plantas. La solución de lixiviación se evaporó, y los precipitados de la muestra se redisolvieron en ácido nítrico concentrado y se diluyeron en una solución de stock de concentración 2 M.

Las muestras disueltas se analizaron en el Metals, Environmental, and Terrestrial Analytical Laboratory en Arizona State University. Se tomó una alícuota para concentración elemental mediante un espectrómetro de masas de plasma acoplado inductivamente de cuadrupolo Thermo Fisher Scientific iCAP (Q-ICP-MS). El estroncio se separó luego con un Prep*FAST*, un sistema automatizado de cromatografía de intercambio iónico a baja presión [79]. El estroncio se aisló de la matriz de muestra usando la resina de intercambio iónico Sr-Ca suministrada por Elemental Scientific, Inc. (Parte CF-MC-SrCa-1000) y ácido nítrico 5 M ultrapuro (HNO_3_). Cada corte de estroncio del Prep*FAST* se secó en un vaso de precipitado de teflón y se digirió con ácido nítrico concentrado y peróxido de hidrógeno al 30% para eliminar los compuestos orgánicos de la resina. Una vez digeridas, las muestras se secaron nuevamente y se reconstituyeron con ácido nítrico 0,32 M. Usando la información de concentración de Q-ICP-MS, las muestras se diluyeron con ácido nítrico 0,32 M a una concentración constante calculada de 50 ppb Sr.

Las proporciones de isótopos de estroncio radiogénicas se midieron en un espectrómetro de masas de plasma acoplado inductivamente de múltiples colectores Thermo-Finnigan Neptune (MC-ICP-MS). El MC-ICP-MS tiene nueve copas Faraday capaces de medir simultáneamente el haz de iones, y este instrumento se configuró con un sistema de introducción de muestras de alta sensibilidad Apex Q de Elemental Scientific, Inc. con un Elemental Scientific, Inc. 50 o 100 μL/minuto Nebulizador microflujo PFA-ST. Este instrumento tiene siete amplificadores 1011 y tres amplificadores 1012 que pueden designarse para cualquiera de las copas de Faraday.

Los datos se recolectaron midiendo 60 relaciones simultáneas integrando 4.194 segundos cada una. Las muestras se corrigieron para blancos en el pico, y la corrección en línea de las contribuciones de ^84^Kr en ^84^Sr y ^86^Kr en ^86^Sr usando una relación ^83^Kr/^84^Kr de 0.201750 y una relación ^83^Kr/^86^Kr de 0.664533, después de la corrección de sesgo de masa instrumental usando una relación ^88^Sr/ ^86^Sr normalizadora de 8.375209. Las muestras se analizaron en tres sesiones analíticas diferentes. La sensibilidad típica fue >10 V en ^88^Sr con una solución Sr de 50 ppb, con valores de ^83^Kr <0.0001 V. Los voltajes de ^85^Rb para las muestras fueron típicamente <0.004 V debido a las bajas relaciones iniciales de Rb/Sr de las muestras y la purificación química efectiva, pero todos los datos se corrigieron con interferencia usando una relación ^85^Rb/^87^Rb de 2.588960, normalizada a ^88^Sr/^86^Sr como se indicó anteriormente. La relación de valores atípicos de dos desviaciones estándar fuera de la media se eliminó utilizando una rutina de corrección matemática 2D de Matlab escrita por el Dr. Stephen Romaniello, quien ahora se encuentra en University of Tennessee. La precisión de valores ^87^Sr/^86^Sr interna típica de dos errores estándar (SE) fue ~1e-6.

Las secuencias incluyeron los estándares SRM 987 de concentración coincidente. SRM 987 se ejecutó como un estándar de horquillado con un valor medido de ^87^Sr/^86^Sr de 0.710252 ± 0.000026 (2σ, *n*=89). Cada sesión analítica incluyó una secuencia que incorpora el estándar SRM 987 en un rango de concentraciones variables para verificar la exactitud de los valores ^87^Sr/^86^Sr para las muestras; los valores informados están todos por encima del umbral para valores exactos de ^87^Sr/^86^Sr dentro del rango de error de los estándares de horquillado. Además, se ejecutó SRM 987 dopado con calcio hasta una relación de Ca/Sr de 500 para simular la exactitud y precisión de los valores de isótopos en muestras pobremente purificadas con bajos rendimientos. Se ejecutó el SRM 987 a una concentración del 50% dopado a un Ca/Sr de 500 como un estándar de verificación con un valor medido de ^87^Sr/^86^Sr de 0.710253 ± 0.000025 (2σ, *n*=15). El agua de mar de IAPSO (Ocean Scientific International Ltd., Havant, Reino Unido) como estándar de verificación secundaria tuvo un valor medido de 0.709182 ± 0.000010 (2σ, *n*=11) que se encuentra adentro del error del valor publicado de 0.709182 ± 0.000004 [80]. NIST 1400 purificado en paralelo con muestras tuvo un valor medido de 0.713124 ± 0.000023 (2σ, *n*=12), lo cual es similar al valor publicado de 0.713150 ± 0.0000160 [81].

## Métodos analíticos

El análisis de grupos de k-means se utilizó para clasificar los datos observados y publicados ^87^Sr/^86^Sr, UTM y de elevación en grupos en el software estadístico R utilizando los paquetes cluster y ggplot2 [82-84]. El análisis de grupos de k-means es un método de agrupamiento locativo puro no jerárquico iterativo divisivo [85,86] que se ha aplicado al análisis de análisis de isótopos ^87^Sr/^86^Sr biodisponibles [40]. Los grupos se definieron en base a las distancias euclidianas para minimizar el error de suma de cuadrados (SSE), minimizando así la variabilidad dentro de los grupos y maximizando la variabilidad entre grupos. Se realizó un procedimiento de asignación al azar que evalúa los cambios en la SSE global para diferentes niveles de grupos. Se seleccionó una solución de grupo comparando la diferencia en SSE en los datos originales con la SSE media de 1,000 iteraciones aleatorias de los datos (Archivos S1 y S2).

# Resultados y discusión

La Tabla 1 reporta los valores observados y publicados de ^87^Sr/^86^Sr de agua, plantas, fauna y muestras de suelo que se incluyeron en el estudio. Los valores de ^87^Sr/^86^Sr variaron de 0.70432 a 0.70641. Entre las muestras de plantas, las plantas no nativas y no comestibles muestreadas de manera oportunista no proporcionaron valores significativamente diferentes de las plantas comestibles nativas que simulan dietas prehispánicas (Fig S1). Todos los datos de concentración elemental traza generados de Q-ICP-MS (Apéndice S1) y datos de estroncio radiogénico de MC-ICP-MS (Apéndice S2) están disponibles como hojas de cálculo suplementarias.

**Table 1. ^87^Sr/^86^Sr y datos de procedencia de la cuenca de México y muestras de referencia del centro de México.**

| Número de laboratorio | Sitio | Material | ^87^Sr/^86^Sr | UTM-E | UTM-N | Elevación (msnm) | Grupo |
| --- | --- | --- | --- | --- | --- | --- | --- |
| ACL-7409-FT | Tequixquiac, Edo. de México | agua de manantial | 0.70476 | 484002 | 2199133 | 2239 | 1 |
| ACL-7409-UF | Tequixquiac, Edo. de México | agua de manantial | 0.70469 | 484002 | 2199133 | 2239 | 1 |
| ACL-7410-FT | Tequixquiac, Edo. de México | agua de manantial | 0.70462 | 480117 | 2200273 | 2533 | 1 |
| ACL-7410-UF | Tequixquiac, Edo. de México | agua de manantial | 0.70458 | 480117 | 2200273 | 2533 | 1 |
| TU-1S | Tula, Hidalgo | suelo^a^ | 0.70500 | 464348 | 2218555 | 2050 | 1 |
| TU-2S | Tula, Hidalgo | suelo^a^ | 0.70501 | 464348 | 2218555 | 2050 | 1 |
| TU-3S | Tula, Hidalgo | suelo^a^ | 0.70469 | 464348 | 2218555 | 2050 | 1 |
| ACL-9058 | Texcotzingo, Edo. de México | *Opuntia ficus* | 0.70471 | 519433 | 2155797 | 2513 | 2 |
| ACL-9059 | Texcotzingo, Edo. de México | *Dahlia pinnata* | 0.70459 | 519358 | 2155730 | 2504 | 2 |
| ACL-9060 | Texcotzingo, Edo. de México | *Agave* spp. | 0.70464 | 519020 | 2155859 | 2534 | 2 |
| ACL-7374 | Xaltocan, Edo. de México | *Kochia scoparia* | 0.70480 | 495867 | 2178713 | 2239 | 2 |
| ACL-7375 | Xaltocan, Edo. de México | *Poa* spp. | 0.70479 | 495867 | 2178716 | 2239 | 2 |
| ACL-7376 | Xaltocan, Edo. de México | *Poa* spp. | 0.70480 | 495878 | 2178710 | 2239 | 2 |
| ACL-7377 | Xaltocan, Edo. de México | *Chenopodium nuttalliae* | 0.70482 | 495882 | 2178707 | 2239 | 2 |
| ACL-7378 | Xaltocan, Edo. de México | *Chenopodium nuttalliae* | 0.70480 | 495883 | 2178708 | 2239 | 2 |
| ACL-7379 | Xaltocan, Edo. de México | *Chenopodium nuttalliae* | 0.70479 | 495892 | 2178710 | 2239 | 2 |
| ACL-7380 | Xaltocan, Edo. de México | *Avena sativa* | 0.70477 | 495702 | 2178935 | 2238 | 2 |
| ACL-7381 | Xaltocan, Edo. de México | *Helianthus* spp. | 0.70479 | 494737 | 2178926 | 2238 | 2 |
| ACL-7382 | Xaltocan, Edo. de México | *Avena sativa* | 0.70478 | 494837 | 2178943 | 2239 | 2 |
| ACL-7383 | Xaltocan, Edo. de México | *Taraxacum officinale* | 0.70474 | 497854 | 2178943 | 2239 | 2 |
| ACL-7384 | Xaltocan, Edo. de México | *Taraxacum officinale* | 0.70475 | 495063 | 2178959 | 2239 | 2 |
| ACL-7385 | Xaltocan, Edo. de México | *Hordeum vulgare* | 0.70478 | 495346 | 2178978 | 2239 | 2 |
| ACL-7386 | Xaltocan, Edo. de México | *Chenopodium nuttalliae* | 0.70481 | 495347 | 2178979 | 2239 | 2 |
| ACL-7387 | Xaltocan, Edo. de México | *Hordeum vulgare* | 0.70484 | 495423 | 2178904 | 2239 | 2 |
| ACL-7388 | Xaltocan, Edo. de México | *Poa* spp. | 0.70484 | 495637 | 2178998 | 2239 | 2 |
| ACL-7389 | Xaltocan, Edo. de México | *Jaltomata procumbens* | 0.70497 | 495884 | 2178860 | 2239 | 2 |
| ACL-7390 | Xaltocan, Edo. de México | *Poa* spp. | 0.70488 | 495868 | 2178714 | 2239 | 2 |
| ACL-7391 | Xaltocan, Edo. de México | *Poa* spp. | 0.70490 | 495868 | 2178713 | 2239 | 2 |
| ACL-7394 | Xaltocan, Edo. de México | *Helianthus* spp. | 0.70481 | 495846 | 2178692 | 2238 | 2 |
| ACL-7397 | Xaltocan, Edo. de México | *Kochia scoparia* | 0.70482 | 495826 | 2178687 | 2238 | 2 |
| ACL-7399 | Xaltocan, Edo. de México | *Agave* spp. | 0.70471 | 495251 | 2181200 | 2241 | 2 |
| ACL-7400 | Xaltocan, Edo. de México | *Opuntia ficus* | 0.70490 | 495292 | 2181209 | 2242 | 2 |
| 11203 CV C2 N334 E96 11 | Teotihuacán, Edo. de México | *Sylvilagus* spp.^b^ | 0.70459 | 516371 | 2177462 | 2351 | 2 |
| 11145 CV C2 N331 E93 1k | Teotihuacán, Edo. de México | *Sylvilagus* spp.^b^ | 0.70458 | 516371 | 2177462 | 2351 | 2 |
| 3110 CV C1 N342 E94 1a | Teotihuacán, Edo. de México | *Sylvilagus* spp.^b^ | 0.70468 | 516371 | 2177462 | 2351 | 2 |
| 8186 CV T N333 E81 2d | Teotihuacán, Edo. de México | *Sylvilagus* spp.^b^ | 0.70459 | 516371 | 2177462 | 2351 | 2 |
| 3294 CV C1 N338 E91 1a | Teotihuacán, Edo. de México | *Sylvilagus* spp.^b^ | 0.70464 | 516371 | 2177462 | 2351 | 2 |
| 7531 CV NS N334 E91 1a | Teotihuacán, Edo. de México | *Sylvilagus* spp.^b^ | 0.70471 | 516371 | 2177462 | 2351 | 2 |
| 22422 CP C5 N348 E116 1f/2a | Teotihuacán, Edo. de México | *Sylvilagus* spp.^b^ | 0.70461 | 516371 | 2177462 | 2351 | 2 |
| 790 CB N325 E16 S | Teotihuacán, Edo. de México | *Sylvilagus* spp.^b^ | 0.70470 | 516371 | 2177462 | 2351 | 2 |
| 706 CB N332 E31 S | Teotihuacán, Edo. de México | *Sylvilagus* spp.^b^ | 0.70465 | 516371 | 2177462 | 2351 | 2 |
| 67145s | Teotihuacán, Edo. de México | suelo^a^ | 0.70435 | 516371 | 2177462 | 2351 | 2 |
| 67145s | Teotihuacán, Edo. de México | suelo^a^ | 0.70432 | 516371 | 2177462 | 2351 | 2 |
| 25166s | Teotihuacán, Edo. de México | suelo^a^ | 0.70438 | 516371 | 2177462 | 2351 | 2 |
| 25166s | Teotihuacán, Edo. de México | suelo^a^ | 0.70441 | 516371 | 2177462 | 2351 | 2 |
| ACL-9046 | Cuicuilco, Cd. de México | *Agave* spp. | 0.70507 | 480790 | 2134234 | 2290 | 3 |
| ACL-9047 | Cuicuilco, Cd. de México | *Dahlia pinnata* | 0.70536 | 480998 | 2134251 | 2288 | 3 |
| ACL-9048 | Cuicuilco, Cd. de México | *Verbascum giganteum* | 0.70502 | 481044 | 2134137 | 2283 | 3 |
| ACL-9049 | Cuicuilco, Cd. de México | *Opuntia ficus* | 0.70591 | 480991 | 2134066 | 2286 | 3 |
| ACL-9050 | Tezozomoc, Cd. de México | *Schinus molle* | 0.70520 | 477880 | 2156155 | 2251 | 3 |
| ACL-9051 | Tezozomoc, Cd. de México | *Agave* spp. | 0.70618 | 478014 | 2156268 | 2251 | 3 |
| ACL-9052 | Naucalli, Edo. de México | *Yucca filifera* | 0.70497 | 474873 | 2155369 | 2264 | 3 |
| ACL-9053 | Naucalli, Edo. de México | *Opuntia ficus* | 0.70503 | 475008 | 2155777 | 2264 | 3 |
| ACL-9054 | Naucalli, Edo. de México | *Agave* spp. | 0.70469 | 474863 | 2155595 | 2264 | 3 |
| ACL-9055 | Cerro Moctezuma, Edo. de México | *Arctostaphylos* spp. | 0.70455 | 473040 | 2154358 | 2385 | 3 |
| ACL-9056 | Cerro Moctezuma, Edo. de México | *Agave* spp. | 0.70489 | 472950 | 2154408 | 2397 | 3 |
| ACL-9057 | Cerro Moctezuma, Edo. de México | *Dahlia pinnata* | 0.70471 | 473033 | 2154438 | 2382 | 3 |
| ACL-9061 | Tlatelolco, Cd. de México | *Poa* spp. | 0.70484 | 485501 | 2150723 | 2231 | 3 |
| ACL-9062 | Tlatelolco, Cd. de México | *Yucca filifera* | 0.70483 | 485523 | 2150718 | 2231 | 3 |
| ACL-9063 | Tlatelolco, Cd. de México | *Opuntia ficus* | 0.70496 | 485597 | 2150719 | 2233 | 3 |
| ACL-9064 | Tlatelolco, Cd. de México | *Agave* spp. | 0.70514 | 485543 | 2150786 | 2233 | 3 |
| ACL-9069 | San Pedro Atocpan, Cd. de México | *Opuntia ficus* | 0.70486 | 494693 | 2122995 | 2239 | 3 |
| ACL-9070 | San Pedro Atocpan, Cd. de México | *Poa* spp. | 0.70481 | 494693 | 2122995 | 2239 | 3 |
| ACL-9071 | San Pedro Atocpan, Cd. de México | *Amaranthus hybridus* | 0.70452 | 494693 | 2122995 | 2239 | 3 |
| ACL-9072 | Santiago Tulyehualco, Cd. de México | *Pinus* spp. | 0.70466 | 498319 | 2128955 | 2252 | 3 |
| ACL-9073 | Santiago Tulyehualco, Cd. de México | *Agave* spp. | 0.70455 | 498319 | 2128955 | 2252 | 3 |
| ACL-9074 | Santiago Tulyehualco, Cd. de México | *Agave* spp. | 0.70462 | 498319 | 2128955 | 2252 | 3 |
| ACL-9075 | Cholula, Puebla | *Dorotheanthus* spp. | 0.70543 | 573378 | 2107043 | 2148 | 4 |
| ACL-9076 | Cholula, Puebla | *Opuntia ficus* | 0.70598 | 573421 | 2107130 | 2150 | 4 |
| ACL-9077 | Cholula, Puebla | *Chenopodium nuttalliae* | 0.70575 | 573314 | 2107191 | 2154 | 4 |
| ACL-9078 | Cholula, Puebla | *Agave* spp. | 0.70602 | 573258 | 2107461 | 2157 | 4 |
| ACL-9079 | Cacaxtla, Tlaxcala | *Dahlia pinnata* | 0.70500 | 569529 | 2127993 | 2298 | 4 |
| ACL-9080 | Cacaxtla, Tlaxcala | *Agave* spp. | 0.70553 | 569461 | 2128024 | 2302 | 4 |
| ACL-9081 | Cacaxtla, Tlaxcala | *Quercus* spp. | 0.70525 | 569280 | 2128072 | 2305 | 4 |
| ACL-9082 | Cacaxtla, Tlaxcala | *Agave* spp. | 0.70541 | 569395 | 2127878 | 2309 | 4 |
| ACL-9065 | Xochicalco, Morelos | *Agave* spp. | 0.70641 | 468747 | 2079174 | 1349 | 5 |
| ACL-9066 | Xochicalco, Morelos | *Enterolobium cyclocarpum* | 0.70521 | 468921 | 2079148 | 1329 | 5 |
| ACL-9067 | Xochicalco, Morelos | *Agave* spp. | 0.70600 | 468629 | 2079292 | 1348 | 5 |
| ACL-9068 | Xochicalco, Morelos | *Agave* spp. | 0.70539 | 468872 | 2079236 | 1340 | 5 |

^a^ Datos de muestras de suelo a granel publicados en [15]

^b^ Datos publicados en [12]

El procedimiento de aleatorización indica que una solución de cinco grupos representa la mayor desviación en la SSE global de la aleatoriedad. Los datos no se distribuyen normalmente. Por lo tanto, las medianas y los rangos intercuartiles se utilizan para caracterizar la variabilidad ^87^Sr/^86^Sr dentro de cada grupo, siguiendo a Price y colegas [18] (Tabla 2, Figs 2 y 3). En los casos en que los sitios (Tabla S1) o los grupos tienen menos de tres muestras, se proporcionan rangos simples en lugar de rangos intercuartiles.

**Tabla 2. Medianas ^87^Sr/^86^Sr y rangos intercuartiles para subregiones del centro de México identificadas mediante análisis de grupos de k-means.**

| Grupo | Subregión geográfica | ^87^Sr/^86^Sr mediana | Rango intecuartil ^87^Sr/^86^Sr | | *n* |
| --- | --- | --- | --- | --- | --- |
| 1 | Norte de la Cuenca de México | 0.70469 | 0.70466 - | 0.70488 | 7 |
| 2 | Cuenca de México Noreste | 0.70476 | 0.70464 - | 0.70481 | 38 |
| 3 | Cuenca de México Suroeste | 0.70488 | 0.70470 - | 0.70506 | 22 |
| 4 | Valle Puebla-Tlaxcala | 0.70548 | 0.70537 - | 0.70581 | 8 |
| 5 | Xochicalco | 0.70570 | 0.70535 - | 0.70610 | 4 |

**Fig. 2. Sitios muestreados dentro del centro de México ordenados por membresía de clúster.** La cuenca de México se destaca en verde, y el extinto sistema de lagos de las tierras altas se muestra en azul. Mapa creado por SIPF con datos de mapas vectoriales y rasterizados de Natural Earth [57].

**Fig 3. Medianas y rangos intercuartiles (rellenos) de cada subregión agrupada en la Tabla 2, con puntos de datos individuales superpuestos.** BoM=Cuenca de México, P-T=Puebla-Tlaxcala.

Cada uno de los cinco grupos constituye una subregión cultural y geográficamente distinta dentro del centro de México (Fig 2). El grupo 1 está formado por dos sitios al norte de la cuenca de México. La propia Cuenca de México se divide en dos grupos, el Grupo 2 que compone el noreste de la Cuenca (tres sitios) y el Grupo 3 que forma el suroeste de la Cuenca (siete sitios). El grupo 4 está compuesto por dos sitios en el valle de Puebla-Tlaxcala, y el grupo 5 está compuesto por el sitio de Xochicalco, al sur de la Cuenca de México. En general, los rangos ^87^Sr/^86^Sr de cada grupo se ajustan a las expectativas geológicas. Los grupos de la Cuenca de México tienen los valores más bajos de ^87^Sr/^86^Sr, lo que refleja los orígenes de la Cuenca en el volcanismo Cenozoico [67,87]. Por el contrario, el grupo Xochicalco tiene los valores ^87^Sr/^86^Sr más altos, lo que indica los orígenes mesozoicos de la región [87,88], aunque la variabilidad intrarregional está limitada debido al número de puntos de datos (*n*=4). Finalmente, el grupo del Valle Puebla-Tlaxcala tiene valores intermedios consistentes con las plataformas mesozoicas de la región cubiertas por rocas volcánicas cenozoicas [89].

Aunque el modelo de cinco grupos divide la Cuenca en dos grupos distintos, es notable que exista una superposición significativa en los valores ^87^Sr/^86^Sr entre los grupos de la Cuenca, así como con los valores ^87^Sr/^86^Sr en el grupo al norte de la Cuenca (Fig 3). Curiosamente, los valores ^87^Sr/^86^Sr del grupo de la Cuenca suroeste de México son más variables dentro de la Cuenca de México. Esto puede reflejar la mayor diversidad en la edad del sustrato geológico, ya que la cuenca del sudoeste está formada por algunas de las formaciones geológicas más antiguas y más jóvenes de la cuenca, incluida la formación Xochitepec (Oligocene, 33.9-23.0 Ma) y la cordillera Chichinautzin. (Cuaternario, 2.6 Ma-presente). A pesar de los rangos superpuestos entre los grupos de la Cuenca de México, los rangos intercuartiles ^87^Sr/^86^Sr indican que los sitios en la Cuenca de México se distinguen fácilmente de los del Valle Puebla-Tlaxcala al este, así como Xochicalco al sur. Por lo tanto, los isótopos de estroncio radiogénico sí se pueden utilizar para abordar cuestiones de paleomovilidad a nivel regional en el centro de México.

El rango intercuartil generado para la Cuenca de México es consistente con rangos publicados previamente. Los dos grupos de la Cuenca de México (2-3) tienen un rango intercuartil combinado de ^87^Sr/^86^Sr=0.70465-0.70487 (*n*=60). Aunque este rango se conforma con el rango de ^87^Sr/^86^Sr=0.7046-0.7051 (*n*=86) publicado por Price y colegas [18], el examen de los rangos intercuartiles ^87^Sr/^86^Sr específicos del sitio indica que este rango local oculta una gran variabilidad dentro de la Cuenca. Muchos sitios en la Cuenca de México aún se pueden distinguir usando análisis de estroncio radiogénico (Fig 4, Tabla S1). Además, con algunas excepciones notables, como Teotihuacán en el grupo de la Cuenca del noreste y Cuicuilco y Tezozomoc en el grupo de la Cuenca del suroeste, todos los rangos "locales" específicos de sitio son más estrechos que los rangos ^87^Sr/^86^Sr de sus grupos asignados. Esto sugiere que aunque el análisis de grupos de k-means es útil a mayor escala para distinguir isotópicamente la Cuenca de México de las regiones circundantes dentro del centro de México, no funciona bien al dividir la Cuenca en subregiones isotópicamente distintas.

**Fig 4. Rangos intercuartiles ^87^Sr/^86^Sr de sitios del centro de México, sombreados por grupo.**

Los puntos de datos individuales están superpuestos. TQX=Tequixquiac, TUL=Tula, TEO=Teotihuacán, TZG=Texcotzingo, XAL=Xaltocan, ATO=San Pedro Atocpan, CMZ=Cerro Moctezuma, CUI=Cuicuilco, NAU=Naucalli, TEZ=Tezozomoc, TLC=Tlatelolco, THY=Santiago Tulyehualco, CHL=Cholula, CXT=Cacaxtla, XCL=Xochicalco.

En el contexto de los estudios de paleomovilidad, la escala de la pregunta de investigación debe determinar si se usan rangos intercuartiles ^87^Sr/^86^Sr agrupados (Tabla 2) o específicos de sitio (Tabla S1) como referencias para el estroncio biodisponible "local". Por ejemplo, si un estudio busca identificar a personas que emigraron a la Cuenca de México desde el centro de México y más allá, el uso de los rangos “locales” de ^87^Sr/^86^Sr en grupo proporciona un mecanismo robusto para establecer individuos como no locales dentro de la Cuenca de México. Sin embargo, si un estudio busca identificar la movilidad residencial de un individuo dentro de la Cuenca de México, el uso de rangos “locales” de ^87^Sr/^86^Sr específicos del sitio proporcionará un análisis de mayor resolución. Con todos estos análisis, es importante tener en cuenta que los valores ^87^Sr/^86^Sr no son únicos y pueden enmascarar la presencia de personas no locales si estos individuos provenían de una región con valores ^87^Sr/^86^Sr parecidas. Por esta razón, el uso de múltiples líneas de evidencia y sistemas isotópicos es esencial [13,51,90].

# Conclusión

El análisis de las proporciones de isótopos de estroncio radiogénicos biodisponibles presentados y publicados del centro de México indica que la Cuenca de México se puede distinguir isotópicamente de las regiones vecinas del centro de México. Además, muchos sitios dentro de la Cuenca de México se pueden distinguir entre sí utilizando isótopos de estroncio radiogénicos, a pesar de cierta superposición en los rangos “locales” de ^87^Sr/^86^Sr a nivel de grupo. Esto indica que los isótopos de estroncio radiogénicos siguen siendo una herramienta poderosa para examinar la paleomovilidad en el centro de México, particularmente si se usan en concierto con otros sistemas isotópicos, como el oxígeno (*δ*^18^O) [91].

Ampliar el conocimiento de la variabilidad del isótopo de estroncio radiogénico en el centro de México es esencial para el futuro trabajo de paleomovilidad en la región, particularmente dada la hipotética importancia de la migración en el desarrollo cultural de la región [3,92]. El trabajo futuro se centrará en aumentar los datos de referencia presentados aquí con muestras de sitios adicionales en todo el centro de México. Estos datos se almacenarán en una base de datos integral de acceso abierto de isótopos de estroncio en todo el centro de México con el objetivo final de desarrollar un isoscape ^87^Sr/^86^Sr para la región.

# Agradecimientos

Agradecemos a Andrés Mejía-Ramón, Dr. Christopher Morehart, Camila Pacheco-Forés y Edgar Paredes por su ayuda con la recolección de muestras en México. En el Archaeological Chemistry Laboratory, estamos agradecidos con los aprendices de investigación Aimee Alvarado, Jorge Benavente, Sibella Campbell, Eric Flores, Zen García, Kari Guilbault, Arman Gurule, Sparshee Naik, Elizabeth Rausch, Emily Steinberg, Alyssa Torres, Rebecca Ulloa y Tajinder Virdee. En el Metals, Environmental, and Terrestrial Analytical Laboratory estamos agradecidos por la asistencia del Dr. Stephen Romaniello, el Dr. Trevor Martin y Natasha Zolotova. Le agradecemos a la Dra. Christina Stantis y a un revisor anónimo por proporcionar comentarios perspicaces que mejoraron la claridad del manuscrito.

# Referencias Citadas

1. Millon R. Urbanization at Teotihuacan, Mexico. Austin, TX: University of Texas Press; 1973.
2. Spence MW. Tlailotlacan, a Zapotec enclave in Teotihuacan. In: Berlo JC, editor. Art, ideology, and the city of Teotihuacan. Washington, D.C.: Dumbarton Oaks; 1992. pp. 59–88.
3. Beekman CS, Christensen AF. Controlling for doubt and uncertainty through multiple lines of evidence: a new look at the Mesoamerican Nahua migrations. J Archaeol Method Theory. 2003;10: 111–164. doi:10.1023/A:1024519712257
4. Begun EM. Detecting ethnicity at Teotihuacan through archaeology: the West Mexican presence at Structure N1W5:19. Ph.D. dissertation, University of Iowa. 2013.
5. Christensen AF. Cranial non-metric variation in north and central Mexico. Anthropol Anz. 1997;55: 15–32.
6. González-José R, Martínez-Abadías N, González-Martín A, Bautista-Martínez J, Gómez-Valdés J, Quinto M, et al. Detection of a population replacement at the Classic–Postclassic transition in Mexico. Proc R Soc B Biol Sci. 2007;274: 681–688. doi:10.1098/rspb.2006.0151
7. Ragsdale CS. Regional population structure in Postclassic Mexico. Anc Mesoam. 2017; 1–13. doi:10.1017/S0956536117000013
8. Ragsdale CS, Edgar HJH. Population continuity and replacement in the pre-contact Valley of Mexico. In: Willermet C, Cucina A, editors. Bioarchaeology of pre-Columbian Mesoamerica: an interdisciplinary approach. Gainesville, FL: University Press of Florida; 2018. pp. 37–69.
9. Meza-Peñaloza A, Zertuche F, García-Velasco M, Morehart C. A non-metric traits study of skulls from Epiclassic Xaltocan in relation to other Mesoamerican cultures. J Archaeol Sci Rep. 2019;23: 559–566. doi:10.1016/j.jasrep.2018.11.031
10. Kemp BM, Reséndez A, Román Berrelleza JA, Malhi RS, Smith DG. An analysis of ancient Aztec mtDNA from Tlatelolco: pre-Columbian relations and the spread of Uto-Aztecan. In: Reed DM, editor. Biomolecular archaeology: genetic approaches to the past. Carbondale, IL: Southern Illinois University Press; 2005. pp. 22–46.
11. Aguirre-Samudio A, González-Sobrino BZ, Álvarez-Sandoval BA, Montiel R, Serrano-Sánchez C, Meza-Peñaloza A. Genetic history of Classic period Teotihuacan burials in central Mexico. Rev Argent Antropol Biológica. 2017;19: 7–20.
12. Price TD, Manzanilla L, Middleton WD. Immigration and the ancient city of Teotihuacan in Mexico: a study using strontium isotope ratios in human bone and teeth. J Archaeol Sci. 2000;27: 903–913. doi:10.1006/jasc.1999.0504
13. White CD, Price TD, Longstaffe FJ. Residential histories of the human sacrifices at the Moon Pyramid, Teotihuacan: evidence from oxygen and strontium isotopes. Anc Mesoam. 2007;18: 159–172. doi:10.1017/S0956536107000119
14. Manzanilla LR, Mejía G, Jiménez G, Schaaf P, Lailson B, Solís G, et al. Caracterización de la población multiétnica de Teopancazco por isótopos estables, isótopos de estroncio y elementos traza. In: Manzanilla LR, editor. Estudios arqueométricos del centro de barrio de Teopancazco en Teotihuacan. Mexico City: Instituto de Investigaciones Antropológicas, Universidad Nacional Autónoma de México; 2012. pp. 449–465.
15. Schaaf P, Solís G, Manzanilla LR, Hernández T, Lailson B, Horn P. Isótopos de estroncio aplicados a estudios de migración humana en el centro de barrio de Teopancazco, Teotihuacan. In: Manzanilla LR, editor. Estudios arqueométricos del centro de barrio de Teopancazco en Teotihuacan. Mexico City: Instituto de Investigaciones Antropológicas, Universidad Nacional Autónoma de México; 2012. pp. 425–448.
16. Nado KL. Dietary practices, socioeconomic status, and social mobility at Teotihuacan, Mexico. Ph.D. dissertation, Arizona State University. 2017.
17. Solís Pichardo G, Schaaf P, Hernández Treviño T, Lailson B, Manzanilla LR, Horn P. Migrants in Teopancazco: evidence from strontium isotopic studies. In: Manzanilla LR, editor. Multiethnicity and migration at Teopancazco: investigations of a Teotihuacan neighborhood center. Gainesville, FL: University Press of Florida; 2017. pp. 143–163.
18. Price TD, Burton JH, Fullagar PD, Wright LE, Buikstra JE, Tiesler V. Strontium isotopes and the study of human mobility in ancient Mesoamerica. Lat Am Antiq. 2008;19: 167–180. doi:10.2307/25478222
19. Luz B, Kolodny Y, Horowitz M. Fractionation of oxygen isotopes between mammalian bone-phosphate and environmental drinking water. Geochim Cosmochim Acta. 1984;48: 1689–1693. doi:10.1016/0016-7037(84)90338-7
20. Ericson JE. Strontium isotope characterization in the study of prehistoric human ecology. J Hum Evol. 1985;14: 503–514.
21. Schwarcz HP, Gibbs L, Knyf M. Oxygen isotope analysis as an indicator of place of origin. Snake Hill: an investigation of a military cemetery from the War of 1812. Toronto, ON: Dundurn Press; 1991. pp. 263–268.
22. Gulson BL, Jameson WW, Gillings BR. Stable lead isotopes in teeth as indicators of past domicile-a potential new tool in forensic science? J Forensic Sci. 1997;42: 787–791.
23. Ehleringer JR, Bowen GJ, Chesson LA, West AG, Podlesak DW, Cerling TE. Hydrogen and oxygen isotope ratios in human hair are related to geography. Proc Natl Acad Sci. 2008;105: 2788–2793.
24. Faure G, Powell JL. Strontium isotope geology. New York, NY: Springer-Verlag; 1972.
25. Turekian KK, Kulp JL. Strontium content of human bones. Science. 1956;124: 405–407.
26. Sealy JC, van der Merwe NJ, Sillen A, Kruger FJ, Krueger HW. ^87^Sr/^86^Sr as a dietary indicator in modern and archaeological bone. J Archaeol Sci. 1991;18: 399–416. doi:10.1016/0305-4403(91)90074-Y
27. Blum JD, Taliaferro EH, Weisse MT, Holmes RT. Changes in Sr/Ca, Ba/Ca and ^87^Sr/^86^Sr ratios between trophic levels in two forest ecosystems in the northeastern U.S.A. Biogeochemistry. 2000;49: 87–101.
28. Flockhart DTT, Kyser TK, Chipley D, Miller NG, Norris DR. Experimental evidence shows no fractionation of strontium isotopes (^87^Sr/^86^Sr) among soil, plants, and herbivores: implications for tracking wildlife and forensic science. Isotopes Environ Health Stud. 2015;51: 372–381. doi:10.1080/10256016.2015.1021345
29. Price TD, Johnson CM, Ezzo JA, Ericson J, Burton JH. Residential mobility in the prehistoric southwest United States: a preliminary study using strontium isotope analysis. J Archaeol Sci. 1994;21: 315–330.
30. Sealy J, Armstrong R, Schrire C. Beyond lifetime averages: tracing life histories through isotopic analysis of different calcified tissues from archaeological human skeletons. Antiquity. 1995;69: 290–300. doi:10.1017/S0003598X00064693
31. Knudson KJ, Stanish C, Lozada Cerna MC, Faull KF, Tantaleán H. Intra-individual variability and strontium isotope measurements: A methodological study using ^87^Sr/^86^Sr data from Pampa de los Gentiles, Chincha Valley, Peru. J Archaeol Sci Rep. 2016;5: 590–597. doi:10.1016/j.jasrep.2016.01.016
32. Faure G. Principles and applications of geochemistry: a comprehensive textbook for geology students. Prentice Hall; 1998.
33. Sillen A, Sealy JC. Chemistry and paleodietary research: no more easy answers. Am Antiq. 1989;54: 504–512.
34. Bentley RA. Strontium isotopes from the earth to the archaeological skeleton: a review. J Archaeol Method Theory. 2006;13: 135–187. doi:10.1007/s10816-006-9009-x
35. Burton JH, Price TD. The use and abuse of trace elements for paleodietary research. Biogeochemical approaches to paleodietary analysis. Springer; 2002. pp. 159–171.
36. Reynolds AC, Quade J, Betancourt JL. Strontium isotopes and nutrient sourcing in a semi-arid woodland. Geoderma. 2012;189–190: 574–584. doi:10.1016/j.geoderma.2012.06.029
37. Price TD, Burton JH, Bentley RA. The characterization of biologically available strontium isotope ratios for the study of prehistoric migration. Archaeometry. 2002;44: 117–135. doi:10.1111/1475-4754.00047
38. Bentley RA, Price TD, Stephan E. Determining the ‘local’ ^87^Sr/^86^Sr range for archaeological skeletons: a case study from Neolithic Europe. J Archaeol Sci. 2004;31: 365–375. doi:10.1016/j.jas.2003.09.003
39. Evans JA, Tatham S. Defining ‘local signature’ in terms of Sr isotope composition using a tenth- to twelfth-century Anglo-Saxon population living on a Jurassic clay-carbonate terrain, Rutland, UK. Geol Soc Lond Spec Publ. 2004;232: 237–248. doi:10.1144/GSL.SP.2004.232.01.21
40. Hodell DA, Quinn RL, Brenner M, Kamenov G. Spatial variation of strontium isotopes (^87^Sr/^86^Sr) in the Maya region: a tool for tracking ancient human migration. J Archaeol Sci. 2004;31: 585–601. doi:10.1016/j.jas.2003.10.009
41. Wright LE. Identifying immigrants to Tikal, Guatemala: defining local variability in strontium isotope ratios of human tooth enamel. J Archaeol Sci. 2005;32: 555–566. doi:10.1016/j.jas.2004.11.011
42. Pestle WJ, Simonetti A, Curet LA. ^87^Sr/^86^Sr variability in Puerto Rico: geological complexity and the study of paleomobility. J Archaeol Sci. 2013;40: 2561–2569. doi:10.1016/j.jas.2013.01.020
43. Knudson KJ, Webb E, White C, Longstaffe FJ. Baseline data for Andean paleomobility research: a radiogenic strontium isotope study of modern Peruvian agricultural soils. Archaeol Anthropol Sci. 2014;6: 205–219. doi:10.1007/s12520-013-0148-1
44. Spence MW, White CD. Mesoamerican bioarchaeology: past and future. Anc Mesoam. 2010;20: 233–240. doi:10.1017/S0956536109990083
45. Price TD, Nakamura S, Suzuki S, Burton JH, Tiesler V. New isotope data on Maya mobility and enclaves at Classic Copan, Honduras. J Anthropol Archaeol. 2014;36: 32–47. doi:10.1016/j.jaa.2014.02.003
46. Price TD, Burton JH, Fullagar PD, Wright LE, Buikstra JE, Tiesler V. Strontium isotopes and the study of human mobility among the ancient Maya. In: Cucina A, editor. Archaeology and Bioarchaeology of Population Movement among the Prehispanic Maya. Springer International Publishing; 2015. pp. 119–132. doi:10.1007/978-3-319-10858-2_11
47. Wright LE. Immigration to Tikal, Guatemala: evidence from stable strontium and oxygen isotopes. J Anthropol Archaeol. 2012;31: 334–352. doi:10.1016/j.jaa.2012.02.001
48. Miller KA. Family, ‘foreigners’, and fictive kinship: a bioarchaeological approach to social organization at Late Classic Copan. Ph.D. dissertation, Arizona State University. 2015.
49. Wrobel GD, Freiwald C, Michael A, Helmke C, Awe J, Kennett DJ, et al. Social identity and geographic origin of Maya burials at Actun Uayazba Kab, Roaring Creek Valley, Belize. J Anthropol Archaeol. 2017;45: 98–114. doi:10.1016/j.jaa.2016.11.004
50. Miller Wolf KA, Freiwald C. Re-interpreting ancient Maya mobility: a strontium isotope baseline for western Honduras. J Archaeol Sci Rep. 2018;20: 799–807. doi:10.1016/j.jasrep.2018.04.023
51. Price TD, Burton JH, Wright LE, White CD, Longstaffe FJ. Victims of sacrifice: isotopic evidence for place of origin. In: Tiesler V, Cucina A, editors. Human sacrifice and ritual body treatments in ancient Maya society. New York: Springer; 2007. pp. 263–292.
52. Thornton EK. Reconstructing ancient Maya animal trade through strontium isotope (^87^Sr/^86^Sr) analysis. J Archaeol Sci. 2011;38: 3254–3263. doi:10.1016/j.jas.2011.06.035
53. Sharpe AE, Emery KF, Inomata T, Triadan D, Kamenov GD, Krigbaum J. Earliest isotopic evidence in the Maya region for animal management and long-distance trade at the site of Ceibal, Guatemala. Proc Natl Acad Sci. 2018;115: 3605–3610. doi:10.1073/pnas.1713880115
54. Thibodeau AM, Luján LL, Killick DJ, Berdan FF, Ruiz J. Was Aztec and Mixtec turquoise mined in the American Southwest? Sci Adv. 2018; 9.
55. Price TD, Tiesler V, Burton JH. Early African diaspora in colonial Campeche, Mexico: strontium isotopic evidence. Am J Phys Anthropol. 2006;130: 485–490. doi:10.1002/ajpa.20390
56. Price TD, Burton JH, Cucina A, Zabala P, Frei R, Tykot Robert H, et al. Isotopic studies of human skeletal remains from a sixteenth to seventeenth century AD churchyard in Campeche, Mexico: diet, place of origin, and age. Curr Anthropol. 2012;53: 396–433. doi:10.1086/666492
57. Natural Earth. [cited 3 Feb 2020]. Available: <https://www.naturalearthdata.com/>
58. Servicio Geológico Mexicano. Carta geológica de la República Mexicana. Pachuca, Hidalgo: Servicio Geológico Mexicano; 2007.
59. de Cserna Z. An outline of the geology of Mexico. In: Bally AW, Palmer AR, editors. The geology of North America--an overview. Boulder, CO: Geological Society of America; 1989. pp. 233–264.
60. Ferrusquía-Villafranca I. Geology of Mexico: a synopsis. In: Ramamoorthy TP, Bye R, Lot A, Fa J, editors. Biological diversity of Mexico: origins and distribution. New York: Oxford University Press; 1993. pp. 3–107.
61. Morán-Zenteno D. The geology of the Mexican republic. Cincinnati, OH: American Association of Petroleum Geologists; 1994.
62. Ohmoto H, Hart SR, Holland HD. Studies in the Providencia area, Mexico, II, K-Ar and Rb-Sr ages of intrusive rocks and hydrothermal minerals. Econ Geol. 1966;61: 1205–1213.
63. Mooser F. Historia geológica de la cuenca de México. Memoria de las obras del sistema de drenaje profundo del Distrito Federal. Mexico City: Departamento del Distrito Federal; 1975. pp. 7–38.
64. Moorbath S, Thorpe RS, Gibson IL. Strontium isotope evidence for petrogenesis of Mexican andesites. Nature. 1978;271: 437–439. doi:10.1038/271437a0
65. Demant A. Interpretación geodinámica del volcanismo del Eje Neovolcánico Transmexicano. Rev Mex Cienc Geológicas. 1981;5: 217–222.
66. Torres-Alvarado IS, Verma SP, Carrasco-Núñez G. Compilation of radiogenic isotope data in Mexico and their petrogenetic implications. J Earth Syst Sci. 2000;109: 67–78. doi:10.1007/BF02719150
67. Vázquez-Sánchez E, Jaimes-Palomera R. Geología de la cuenca de México. Geofísica Int. 1989;28: 133–190.
68. Hoogewerff J, Papesch W, Kralik M, Berner M, Vroon P, Miesbauer H, et al. The last domicile of the Iceman from Hauslabjoch: a geochemical approach using Sr, C and O isotopes and trace element signatures. J Archaeol Sci. 2001;28: 983–989.
69. Aubert D, Probst A, Stille P, Viville D. Evidence of hydrological control of Sr behavior in stream water (Strengbach catchment, Vosges mountains, France). Appl Geochem. 2002;17: 285–300. doi:10.1016/S0883-2927(01)00080-4
70. Tricca A, Stille P, Steinmann M, Kiefel B, Samuel J, Eikenberg J. Rare earth elements and Sr and Nd isotopic compositions of dissolved and suspended loads from small river systems in the Vosges mountains (France), the river Rhine and groundwater. Chem Geol. 1999;160: 139–158.
71. Dasch EJ. Strontium isotopes in weathering profiles, deep-sea sediments, and sedimentary rocks. Geochim Cosmochim Acta. 1969;33: 1521–1552.
72. Budd P, Montgomery J, Barreiro B, Thomas RG. Differential diagenesis of strontium in archaeological human dental tissues. Appl Geochem. 2000;15: 687–694. doi:10.1016/S0883-2927(99)00069-4
73. Négrel Ph, Petelet-Giraud E, Barbier J, Gautier E. Surface water–groundwater interactions in an alluvial plain: chemical and isotopic systematics. J Hydrol. 2003;277: 248–267. doi:10.1016/S0022-1694(03)00125-2
74. Palmer MR, Edmond JM. Controls over the strontium isotope composition of river water. Geochim Cosmochim Acta. 1992;56: 2099–2111. doi:10.1016/0016-7037(92)90332-D
75. Grimstead DN, Nugent S, Whipple J. Why a standardization of strontium isotope baseline environmental data is needed and recommendations for methodology. Adv Archaeol Pract. 2017;5: 184–195. doi:10.1017/aap.2017.6
76. Knudson KJ. Oxygen isotope analysis in a land of environmental extremes: the complexities of isotopic work in the Andes. Int J Osteoarchaeol. 2009;19: 171–191. doi:10.1002/oa.1042
77. Buzon MR, Conlee CA, Bowen GJ. Refining oxygen isotope analysis in the Nasca region of Peru: an investigation of water sources and archaeological samples. Int J Osteoarchaeol. 2011;21: 446–455. doi:10.1002/oa.1151
78. Runia LexT. Strontium and calcium distribution in plants: effect on palaeodietary studies. J Archaeol Sci. 1987;14: 599–608. doi:10.1016/0305-4403(87)90078-1
79. Romaniello SJ, Field MP, Smith HB, Gordon GW, Kim MH, Anbar AD. Fully automated chromatographic purification of Sr and Ca for isotopic analysis. J Anal At Spectrom. 2015;30: 1906–1912. doi:10.1039/C5JA00205B
80. Ma J, Wei G, Liu Y, Ren Z, Xu Y, Yang Y. Precise measurement of stable (*δ*^88/86^Sr) and radiogenic (^87^Sr/^86^Sr) strontium isotope ratios in geological standard reference materials using MC-ICP-MS. Chin Sci Bull. 2013;58: 3111–3118. doi:10.1007/s11434-013-5803-5
81. Galler P, Limbeck A, Boulyga SF, Stingeder G, Hirata T, Prohaska T. Development of an on-line flow injection Sr/matrix separation method for accurate, high-throughput determination of Sr isotope ratios by multiple collector-inductively coupled plasma-mass spectrometry. Anal Chem. 2007;79: 5023–5029. doi:10.1021/ac070307h
82. Wickham H. ggplot2: elegant graphics for data analysis. New York: Springer-Verlag; 2009.
83. R Core Team. R: a language and environment for statistical computing. Vienna, Austria: R Foundation for Statistical Computing; 2016.
84. Maechler M, Rousseeuw P, Struyf A, Hubert M, Hornik K. cluster: cluster analysis basics and extensions. R package version 2.0.6; 2017. Available: <https://cran.r-project.org/web/packages/cluster/cluster.pdf>
85. Kintigh KW. Intrasite spatial analysis: a commentary on major methods. In: Voorrips A, editor. Mathematics and information science in archaeology: a flexible framework. Bonn: Holos; 1990. pp. 165–200.
86. Kintigh KW, Ammerman AJ. Heuristic approaches to spatial analysis in archaeology. Am Antiq. 1982;47: 31–63. doi:10.2307/280052
87. Servicio Geológico Mexicano. Distrito Federal, Estado de México y Morelos: carta geológico-minera. Pachuca, Hidalgo: Servicio Geológico Mexicano; 2009.
88. Fries, Jr. C. Geology of the state of Morelos and contiguous areas in south-central Mexico. Ph.D. dissertation, University of Arizona. 1958.
89. Servicio Geológico Mexicano. Estados de Puebla y Tlaxcala: carta geológico-minera. Pachuca, Hidalgo: Servicio Geológico Mexicano; 2008.
90. Knudson KJ, Price TD. Utility of multiple chemical techniques in archaeological residential mobility studies: case studies from Tiwanaku- and Chiribaya-affiliated sites in the Andes. Am J Phys Anthropol. 2007;132: 25–39. doi:10.1002/ajpa.20480
91. Moreiras Reynaga D. The life histories of Aztec sacrifices: a stable isotope study (C, N, and O) of offerings from Tlatelolco and the Templo Mayor of Tenochtitlan. Ph.D. dissertation, University of Western Ontario. 2019. Available: <https://ir.lib.uwo.ca/etd/6448>
92. Cowgill GL. The debated role of migration in the fall of ancient Teotihuacan in central Mexico. In: Baker BJ, Tsuda T, editors. Migration and disruptions: toward a unifying theory of ancient and contemporary migrations. Gainesville, FL: University Press of Florida; 2015. pp. 97–122.

# Información adicional

**Archivo S1. Hoja de cálculo de datos CSV para cargar en R para usar con código en el archivo S2.**

**Archivo S2. Código R para el análisis estadístico de ^87^Sr/^86^Sr, coordenadas UTM y datos de elevación.**

**S1 Fig. Grupos de ^87^Sr/^86^Sr valores en muestras de plantas por origen de planta.** No hubo diferencias significativas entre las plantas nativas comestibles y las plantas nativas no comestibles o plantas no nativas. Si bien las plantas no comestibles nativas y no nativas no habrían contribuido a valores pasados de ^87^Sr/^86^Sr biodisponibles en humanos y animales, se incluyen en este estudio para caracterizar aún más los valores de estroncio biodisponibles en los ecosistemas locales.

**S1 Apéndice. Datos generados de trazas de concentraciónes elementales de Q-ICP-MS en muestras de plantas y agua del centro de México.**

**S2 Apéndice. Datos generados de valores ^87^Sr/^86^Sr de MC-ICP-MS en muestras de plantas y agua del centro de México.**

**S1 Tabla. Medianas y rangos intercuartiles de ^87^Sr/^86^Sr en sitios del centro de México.**
